# Solubility Parameters of Amino Acids on Liquid–Liquid Phase Separation and Aggregation of Proteins

**DOI:** 10.3389/fcell.2021.691052

**Published:** 2021-06-16

**Authors:** Akira Nomoto, Suguru Nishinami, Kentaro Shiraki

**Affiliations:** Pure and Applied Sciences, University of Tsukuba, Tsukuba, Japan

**Keywords:** phase separation, amino acid, solubility, soluble interaction, insoluble interaction

## Abstract

The solution properties of amino acids determine the folding, aggregation, and liquid–liquid phase separation (LLPS) behaviors of proteins. Various indices of amino acids, such as solubility, hydropathy, and conformational parameter, describe the behaviors of protein folding and solubility both *in vitro* and *in vivo*. However, understanding the propensity of LLPS and aggregation is difficult due to the multiple interactions among different amino acids. Here, the solubilities of aromatic amino acids (SAs) were investigated in solution containing 20 types of amino acids as amino acid solvents. The parameters of SAs in amino acid solvents (PSASs) were varied and dependent on the type of the solvent. Specifically, Tyr and Trp had the highest positive values while Glu and Asp had the lowest. The PSAS values represent soluble and insoluble interactions, which collectively are the driving force underlying the formation of droplets and aggregates. Interestingly, the PSAS of a soluble solvent reflected the affinity between amino acids and aromatic rings, while that of an insoluble solvent reflected the affinity between amino acids and water. These findings suggest that the PSAS can distinguish amino acids that contribute to droplet and aggregate formation, and provide a deeper understanding of LLPS and aggregation of proteins.

## Introduction

### Phase Separation of Proteins and Amino Acid Characteristics

Liquid–liquid phase separation (LLPS) of proteins is a phenomenon is characterized by the formation of condensates consisting of homogeneously dispersed proteins with a composition different from that of the bulk material due to a stimulus or environmental change (Uversky, 2017; Boeynaems et al., 2018). Previous studies have reported that various biological reactions occur via phase separation of proteins (Yoshizawa et al., 2020). For example, droplets formed by LLPS are involved in the stress response and the regulation of transcription and translation (Hnisz et al., 2017; Riback et al., 2017; Nosella and Forman-Kay, 2021). Moreover, amyloids formed by liquid–solid phase separation are known to be involved in various diseases (Elbaum-Garfinkle, 2019; Wang and Zhang, 2019). The phase separation of proteins is also valuable for industrial applications, such as the concentration and stabilization of antibodies and enzymes for use as drugs (Kurinomaru et al., 2014; Izaki et al., 2015; Maruyama et al., 2015; Mimura et al., 2019). Furthermore, the phase separation of proteins from the dispersed and redispersed states can be controlled to some extent by small molecular additives (Kurinomaru and Shiraki, 2017; Shiraki et al., 2020).

Recently, a database was constructed to organize the relationships of LLPS with biological reactions (Li et al., 2020; Mészáros et al., 2020; Ning et al., 2020). Proteins that are prone to LLPS are characterized by intrinsically disordered regions (IDR) (Uversky, 2017; Kuechler et al., 2020). The propensity of proteins to form droplets and aggregates, which is defined as the “propensity of phase separation (PPS)” in this paper, can be predicted to some extent by the length and types of amino acids in the IDR (Liu et al., 2019; Schuster et al., 2020). In particular, charged or hydrophilic amino acids with low complexity sequences are involved in the LLPS of proteins (Chong et al., 2018; Das et al., 2020; Kuechler et al., 2020). Therefore, understanding the PPS at the amino acid level is important to elucidate the molecular mechanisms underlying the biological reactions of proteins.

### Amino Acid Index

Various properties of amino acids have been investigated to reveal the nature of protein folding and aggregation (Table 1). The solubility of an amino acid (SA) is the simplest index of the affinity between an amino acid and a solvent (Fleck and Petrosyan, 2014). A study conducted in the 1930s by Dalton and McMeekin on SA in water is probably the first to propose indexing of the properties of amino acids (Amend and Helgeson, 1997). Thereafter, the concepts of hydrophobic, chaotropic, and kosmotropic were established (Relating et al., 1964; Bigelow, 1967; Collins, 1995, 1997). In the 1970s, Nozaki and Tanford (1971) measured the SA in the solution of protein denaturants, which is among the first studies of protein stability in solvents. In the solubility experiments conducted by Fauchere, the transfer free energy of each amino acid between octanol and water was measured and the hydrophobicity of the side chain of each amino acid was indexed by subtracting the transfer free energy of Gly from that of each amino acid (Fauchere et al., 1988). Since the affinity of an amino acid for a solvent is dependent on whether the amino acid is buried inside or exposed on the surface of the protein, Miller indexed the propensity of the internal or external presence of amino acid with the use of crystals structures of proteins (Miller et al., 1987). After the structure of proteins was elucidated, Chou and Fasman indexed the ability of each amino acid to form secondary structures by investigating the amino acids contained in the secondary structures of the proteins (Chou and Fasman, 1974). According to Kyte and Doolittle (1982), hydropathy is the most important index of amino acids. Hydropathy, which has been cited more than 20,000 times over the past 30 years, is a practical index calculated from SA and the position of the amino acid in the protein structure. Recently, Hirano and Kameda (2021) defined aromaphilicity as a new index to evaluate the binding affinity of amino acids to the aromatic rings of carbon materials. This index is applicable to assess the affinity of an amino acid to a particular substance.

**TABLE 1:** Various parameters of amino acid in previous studies.

Amino acids	Solubility in water	Side chain hydrophobicity	In–out propensity	Hydropathy	α-helix propensity	β-sheet propensity	Coil propensity	Aromaphilicity
Tyrosine (Tyr)	0.054	0.96	−0.22	−1.3	0.61	1.29	1.19	0.850
Tryptophan (Trp)	1.32	2.25	0.45	−0.9	1.14	1.19	0.82	1.000
Phenylalanine (Phe)	2.80	1.79	0.67	2.8	1.12	1.28	0.81	0.575
Arginine (Arg)	19.59	−1.01	−1.34	−4.5	0.79	0.90	1.20	0.750
Lysine (Lys)	24.66	−0.99	−2.00	−3.9	1.07	0.74	1.05	0.100
Histidine (His)	4.36	0.13	0.04	−3.2	1.24	0.71	0.92	0.450
Proline (Pro)	130.07	0.72	−0.44	−1.6	0.59	0.62	1.45	0.125
Glycine (Gly)	25.23	0.00	0.06	−0.4	0.53	0.81	1.42	0.000
Alanine (Ala)	16.63	0.31	0.20	1.8	1.45	0.97	0.66	0.025
Serine (Ser)	36.57	−0.04	−0.34	−0.8	0.79	0.72	1.27	0.125
Cysteine (Cys)	2.56	1.54	0.67	2.5	0.77	1.30	1.07	0.200
Methionine (Met)	5.59	1.23	0.71	1.9	1.20	1.67	0.61	0.325
Valine (Val)	5.87	1.22	0.61	4.2	1.14	1.65	0.66	0.150
Leucine (Leu)	2.19	1.70	0.65	3.8	1.34	1.22	0.66	0.125
Isoleucine (Ile)	3.17	1.80	0.74	4.5	1.00	1.60	0.78	0.200
Threonine (Thr)	9.79	0.26	−0.26	−0.7	0.82	1.20	1.05	0.075
Glutamine (Gln)	4.25	−0.22	−0.74	−3.5	1.17	1.23	0.79	0.300
Asparagine (Asn)	2.51	−0.60	−0.69	−3.5	0.73	0.65	1.33	0.200
Glutamic acid (Glu)	0.88	−0.64	−1.09	−3.5	1.53	0.26	0.87	0.050
Aspartic acid (Asp)	0.51	−0.77	−0.72	−3.5	0.98	0.80	1.09	−0.025

*The solubility of amino acids in water (g/100 mL) (Fleck and Petrosyan, 2014). The hydrophobicity of the side chain of amino acids (kcal/mol) (Fauchere et al., 1988). The in–out propensity of amino acids in the crystal structure of proteins (kcal/mol) (Miller et al., 1987). The hydropathy index of amino acids (Kyte and Doolittle, 1982). The α-helix, β-sheet, and random-coil propensity index of amino acids in the tertiary structures of proteins (Chou and Fasman, 1974). The aromaphilicity index of amino acids (Hirano and Kameda, 2021).*

### Indices to Understand the PPS

The amino acid indices listed in Table 1 provide basic information to predict the folding and solubility of proteins (Bigelow, 1967; Dubchak et al., 1995; Bhandari et al., 2020). In addition, the degree of the IDR predicted from the amino acid sequence of a protein is also an important parameter to evaluate the PPS (Obradovic et al., 2003; Dosztányi et al., 2005; Xue et al., 2010). However, it is difficult to predict the PPS with combinations of these parameters because the most important feature of phase separation of proteins is caused by multivalent and dynamic interactions among the side chains of amino acids in an aqueous solution (Boeynaems et al., 2018; Murthy et al., 2019; Sahli et al., 2019). In order to gain a deeper understanding of the PPS, this paper proposes a new index of amino acids that reflects the interactions among amino acids from solubility experiments. For example, it is known that when Arg is present in a solvent, the solubility of solute molecules with aromatic rings increases because of the cation-π interaction between Arg in the solvent and the aromatic molecules of the solute (Arakawa et al., 2008; Hirano et al., 2010a, b; Ariki et al., 2011). In other words, it is the SA as a solute in a solvent containing Arg. Thus, the SA in an amino acid solvent is an index to predict the PPS because the interactions among amino acids influence solubility.

## Subsections Relevant for the Subject

### Materials and Methods

All amino acids, NaH_2_PO_4_, and Na_2_HPO_4_ were obtained from Wako Pure Chemical Industries Ltd. (Osaka, Japan). All chemicals were of reagent grade and used as received. The following procedure was used to measure solubility. To investigate the interaction between two amino acids, aromatic amino acids (AAAs) were prepared in powdered form and were respectively dissolved in 50 mM Na-phosphate buffer (pH 7.0) containing 20 different amino acids as “amino acid solvents”. Here, the maximum concentration of each amino acid solvents varied heavily because the solubility of each amino acid in aqueous solution varied. The suspension was heated at 50°C for 1 h with frequent vortexing to dissolve completely powdered AAAs and then incubated at 25°C for 14 h with frequent vortexing. Subsequently, the suspension was centrifuged at 25°C and 18,800 × *g* for 20 min to obtain a supernatant saturated with AAAs. After appropriate dilution of the supernatant with 50 mM Na-phosphate buffer (pH 7.0), the concentrations of the AAAs were determined by measuring the absorbance at 260 to 280 nm along with an appropriate blank using an ND-1000 spectrophotometer (NanoDrop Technologies, Wilmington, DE, United States). The solubilities were then calculated from the standard curves determined for each AAA. This method has the advantage of allowing accurate quantification of the concentrations of amino acids in mixtures (Nishinami et al., 2018; Hirano et al., 2020).

### Solubility of an Amino Acid in 20 Kinds of Amino Acid Solvents

**FIGURE 1:**
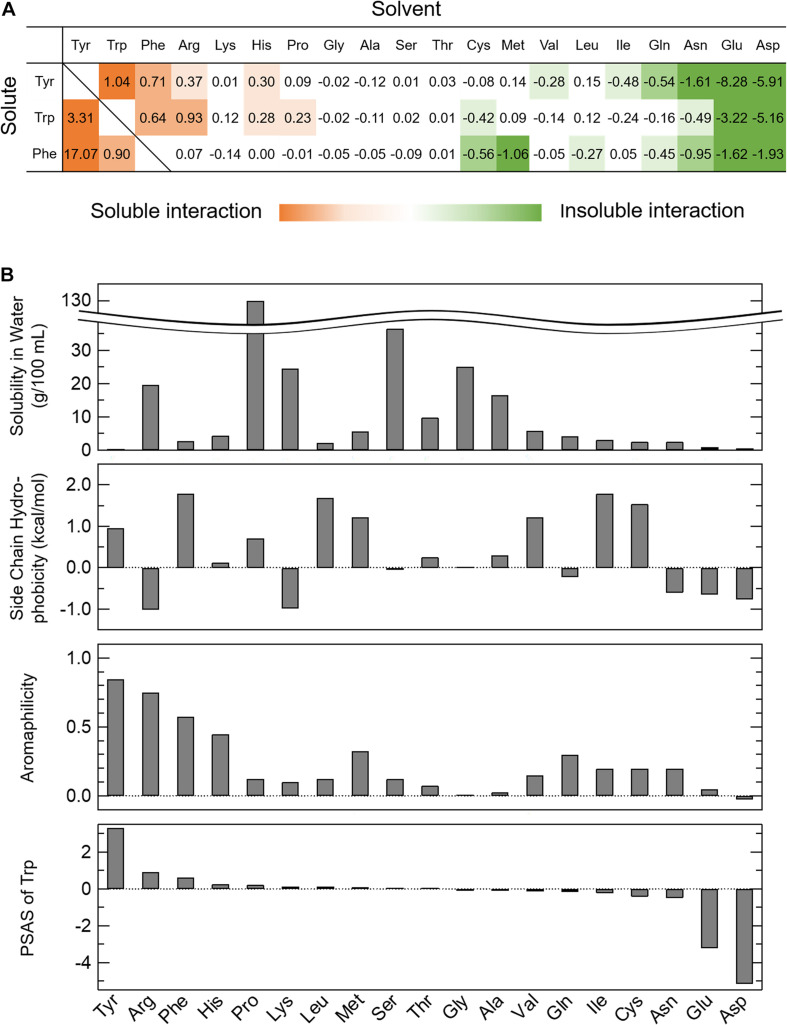
Parameter of solubility of AAAs in amino acid solvents (PSAS). **(A)** The PSAS of AAAs in 20 kinds of amino acid solvents. The PSAS was calculated from Equation (1). The PSAS is shown as a positive value when the solubility of the AAAs increases and as a negative value when it decreases. **(B)** Relationships of the PSAS with the various indices characterizing amino acids. The PSAS of Trp is compared to the solubility in water, side chain hydrophobicity, and aromaphilicity of 20 kinds of amino acids.

The solubilities of AAAs in 20 kinds of amino acid solvents are shown in Supplementary Figure 1A. Based on these results, the parameters of SAs in the amino acid solvents (PSASs) were calculated from a simple Equation (1),

$$\text{PSAS}=\frac{\Delta s}{\Delta c}\ldots \left(1\right)$$

where Δ*s* is the change in the solubility ratio of AAA in the amino acid solvents when the solubility in water is 1 and Δ*c* is the change in the concentration of the amino acid solvent. Briefly, the Equation (1) shows the slope of fitting line in Supplementary Figure 1A. Thus, PSAS indicates the degree to which an amino acid at a concentration of 1 M is present in the solvent solubilizes or insolubilizes the AAA. Some of the amino acids changed the solubility of AAAs at low or high concentrations. Since the concentration at which the effect of solubility changes varied among amino acids, we established the PSAS that did not consider the concentration range of amino acid solvents. A list of PSASs is provided in Figure 1A with solubilized AAAs presented as positive values (red) and insolubilized AAAs as negative values (green). Arg, Pro, and AAA solvents had positive PSAS values, indicating that the interactions between AAAs and these amino acids are favorable to soluble AAAs. However, anionic amino acid solvents had negative PSAS values, indicating that the interactions between AAAs and negatively charged amino acids are unfavorable to soluble AAAs. Unexpectedly, the solvents of Gln, Asn, and hydrophobic amino acid insolubilized AAAs, indicating that interactions between AAAs and these amino acids are favorable, but do not contribute to solubilization of AAAs. When the PSAS value was close to zero, neither soluble nor insoluble effects were at work, suggesting that the interactions among the amino acids were as weak as those with water.

### Comparison of PSAS With Existing Amino Acid Indices

To clarify the relationship between the PSAS proposed in this study and the amino acid indices proposed in previous studies, the PSAS was determined as the balance of the interactions among the amino acids and those of the amino acids and water, as determined by solubility experiments. Therefore, the PSAS was compared with some of the indices listed in Table 1 that reflect affinity for water and the interactions among amino acids and aromatic rings (solubility in water, side chain hydrophobicity, and aromaphilicity). When the PSAS was compared with these indices by row, the order of the PSAS of Trp did not match the solubility in water and side chain hydrophobicity, but it was partially consistent with the aromaphilicity (Figure 1B). The PSAS of Tyr and Phe showed a similar propensity. This result suggested that PSAS reflects the binding affinity of the side chains for aromatic rings rather than the interaction between amino acids and water. However, as the order of PSAS did not completely match the aromaphilicity, the PSAS could not be explained by a simple affinity between side chains and aromatic rings. Next, the PSAS was compared with these indices by column, which showed that the magnitude of the PSAS of the soluble solvents (Trp, Arg, His, and Pro) was in the order of Trp > Tyr > Phe, consistent with the magnitude of aromaphilicity (Figure 1A and Table 1). On the other hand, the magnitude of the PSAS of the insoluble solvents (Gln, Asn, Glu, Asp, Val, and Ile) was in the order of Phe > Trp > Tyr or Trp > Phe > Tyr, which was consistent with the magnitude of solubility in water or side chain hydrophobicity (Figure 1A and Table 1). These results suggest that the soluble interactions reflect the affinities among the amino acids and aromatic rings, while the insoluble interactions reflect the affinities of the amino acids and water. Hence, the PSAS mainly reflects the interactions among the amino acids as a soluble property and those of the amino acids and water as an insoluble property.

### Properties of Gln, Asn, and Pro

Gln, Asn, and Pro are abundant in low complexity regions of proteins that undergo phase separation (Budini et al., 2012; Riback et al., 2017), although the functions of these amino acids are unclear. Gln and Asn insolubilized the AAAs, while Pro solubilized the AAAs (Figure 1A). Based on the PSAS, Gln and Asn can cause phase separation by interacting with AAAs. Molecular dynamics simulation may be able to reveal interactions among amino acids that have not received much attention so far by investigating the PSAS other than AAAs.

On the other hand, it is difficult to understand the PSAS of Pro because the Pro solvent in this experiment system may change the properties of the solution, such as viscosity and polarity, due to the extremely high solubility of Pro (Wu et al., 2001; Zhao, 2006; Ferreira et al., 2019). Therefore, the PSAS of Pro may not be simply comparable to the PSAS of other amino acids. When we investigate the solubility of 20 solute amino acids in 20 kinds of amino acid solvents, it is worthwhile to examine the difference between the solubility change due to the interaction between amino acids and that due to solvent effect.

## Discussion

### Phase Separation of Proteins Based on PSAS

The novel index PSAS categorizes amino acids into soluble and insoluble interactions (Figure 1A). A soluble interaction represented by the PSAS (red area in Figure 1A) indicates that an amino acid is highly soluble in an amino acid solvent. At the microscopic level, there is an attractive interaction of the solute amino acid with the solvent amino acid, meaning that the water molecules are involved in the interaction. In other words, the soluble interaction of the PSAS is considered similar to the intermolecular interactions within a droplet formed by LLPS. Actually, the cation-π and *π*–π interactions, which are known to be the driving forces for droplet formation (e.g., Ddx4 and hnRNPA2) (Nott et al., 2015; Vernon et al., 2018; Dignon et al., 2020), are represented by the PSAS as soluble interactions. On the other hand, the insoluble interaction represented by the PSAS (green area in Figure 1A) indicates that the solute amino acid is insoluble in the amino acid solvent. There are two possible mechanisms underlying the insoluble effect expressed by the PSAS: (i) the repulsive interactions between the solute and solvent amino acids result in precipitation of the solute amino acids because the solute and solvent amino acids are negatively charged at pH 7.0 (e.g., Glu and Asp) and (ii) water molecules are not involved in the attractive interaction between the solute and solvent amino acids, resulting in coprecipitation of the two amino acids (e.g., Gln, Asn, and hydrophobic amino acids). In particular, the insoluble interaction represented by (ii) is the same as the intermolecular interaction of aggregates formed by liquid–solid phase separation (e.g., ovalbumin, lysozyme, and α-synuclein) (Iwashita et al., 2018; Stephens and Kaminski Schierle, 2019). Actually, the hydrophobic interactions, which are known to be the driving force of aggregate formation, were represented by the PSAS as insoluble interactions. Therefore, the soluble interaction expressed by the PSAS may represent droplet formation ability and the insoluble interaction may represent aggregate formation ability.

The PPS of a protein must be considered in the interactions of many kinds of amino acids. However, the PSAS represents only the interaction between the two types of amino acids. Actually, it has been reported that hydrophobic interactions, which are considered the driving force of aggregation, stabilize droplets, as well as electrostatic and *π*–*π* interactions, which are considered the driving forces of droplet formation, stabilize aggregates (e.g., tau and IgG) (Ma et al., 2010; Matsuda et al., 2018; Krainer et al., 2021; Lin et al., 2021). Therefore, it will be difficult to completely distinguish between aggregates and droplets by only focusing on the main interactions. When distinguishing between droplet and aggregate formation abilities based on the PSAS, the properties of the assemblies must be considered from only the interaction between the two types of amino acids.

Finally, for future applications, the PSAS at the amino acid level did not fully match that at the protein level. To more clearly illustrate this finding, the PSAS of Trp was relatively large, but is rarely present in the IDR of proteins that are prone to LLPS (Wang et al., 2014; Martin and Mittag, 2018; Martin et al., 2020), while Gly and Ser had PSAS values close to zero, but are known to be abundant in the IDR of proteins that are prone to LLPS (Schuster et al., 2018; Hardenberg et al., 2021). These conflicts are thought to be due to differences in the properties of free amino acids as solutes and amino acids during peptide formation. These differences indicate the importance of the liquid–liquid or liquid–solid phase separations of proteins. In addition, several methods have been developed to predict the PPS of proteins (Vernon and Forman-Kay, 2019; Hardenberg et al., 2021; van Mierlo et al., 2021). These prediction methods can identify proteins with a high PPS from the proteome, such as the distribution of the amino acid residues and the complexity of the sequence. The PSAS is expected to be a prominent parameter for prediction methods, typically machine learning, due to the experimental interactions between the solute and solvent amino acids, including water.

## Data Availability Statement

The original contributions presented in the study are included in the article/Supplementary Material, further inquiries can be directed to the corresponding author.

## Author Contributions

AN performed all the experiments and wrote the manuscript. SN supervised the experiments. KS revised and edited the manuscript. All authors have read and approved the final manuscript.

## Conflict of Interest

The authors declare that the research was conducted in the absence of any commercial or financial relationships that could be construed as a potential conflict of interest.
