# Supplementary material for: Solubility Parameters of Amino Acids on Liquid–Liquid Phase Separation and Aggregation of Proteins
Source: Front Cell Dev Biol. 2021 Jun 16;9:691052. doi: 10.3389/fcell.2021.691052 (PMC8242209; doi:10.3389/fcell.2021.691052)
Supplement: Supplementary file 1 [file Table_1.docx]

Supplementary Material

## Supplementary Figures

**
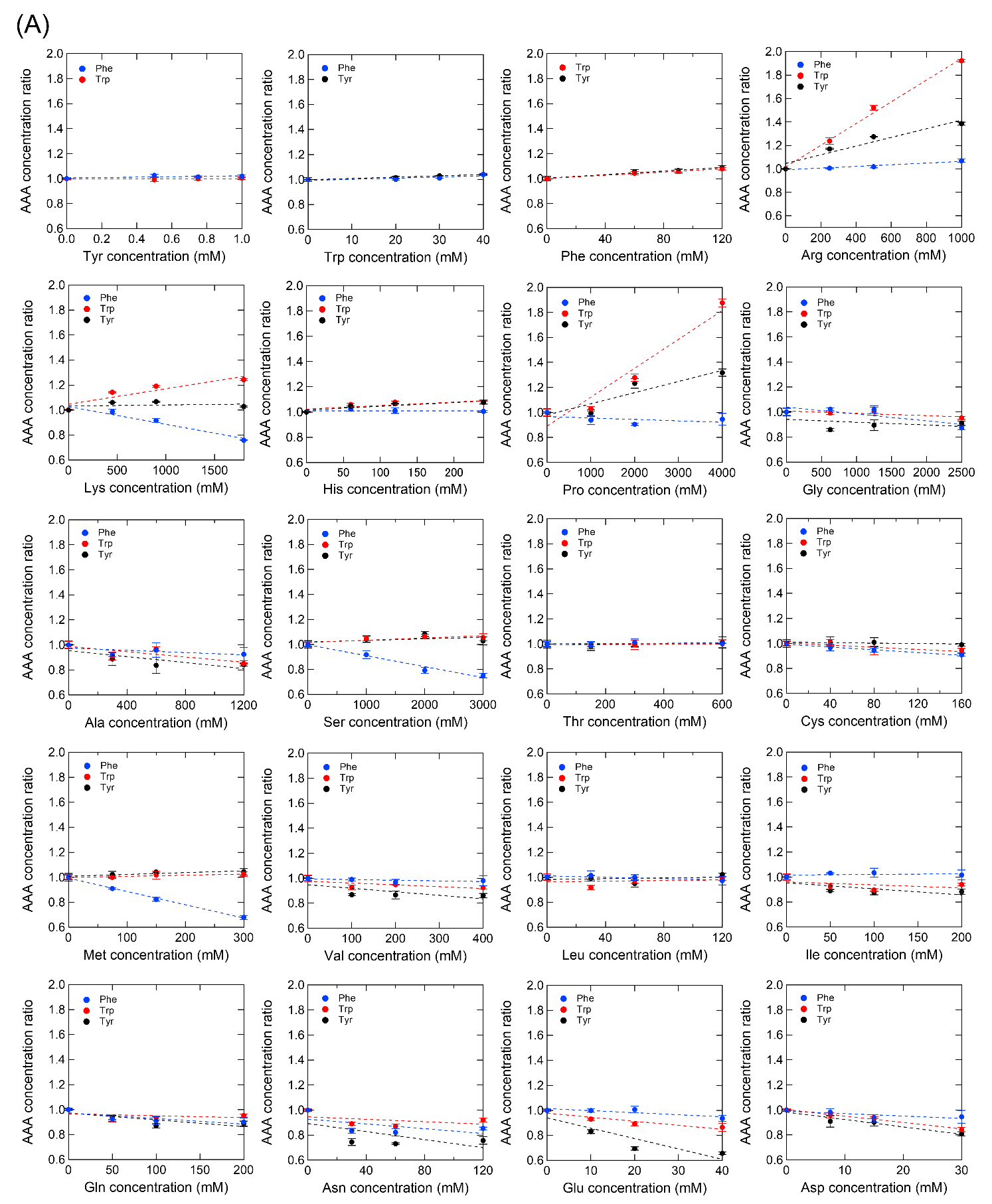
**

**
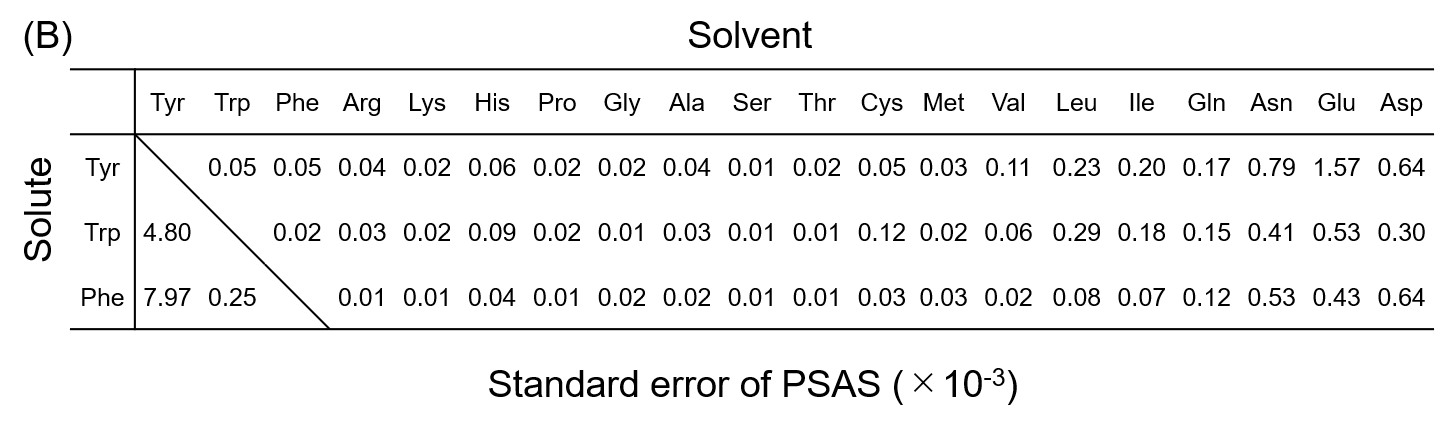
**

**Supplementary Figure 1.** (A) The solubility of AAAs in amino acid solvents. Each sample (Tyr, black; Trp, red; Phe, blue) contains 0–4000 mM amino acids and 50 mM Na-phosphate buffer (pH 7.0). The concentrations of the AAAs (Tyr, Trp, and Phe) in 0 mM amino acid solvents were 2, 65, and 172 mM, respectively. Measurements were performed three times, and the error bars indicate the standard deviation of the mean. (B) The ±standard errors of PSAS were obtained from independent measurements (*n* = 3).
